# Maternity Care Providers’ Experiences with Providing Information on Newborn Bloodspot Screening During Pregnancy: A Dutch Survey Study

**DOI:** 10.3390/ijns11010005

**Published:** 2025-01-08

**Authors:** Jasmijn E. Klapwijk, Janneke Gitsels-van der Wal, Linda Martin, Rendelien K. Verschoof-Puite, Ellen Elsinghorst, Lidewij Henneman

**Affiliations:** 1 Department of Human Genetics and Amsterdam Reproduction and Development Research Institute, Amsterdam UMC, Vrije Universiteit Amsterdam, 1007 MB Amsterdam, The Netherlands; 2 Department of Midwifery Science and Amsterdam Public Health Research Institute, Amsterdam UMC, Vrije Universiteit Amsterdam, 1007 MB Amsterdam, The Netherlands; 3 Midwifery Academy Amsterdam Groningen, InHolland, 1059 GL Amsterdam, The Netherlands; 4 Department for Vaccine Supply and Prevention Programmes, RIVM Dutch National Institute for Public Health and the Environment, 3720 BA Bilthoven, The Netherlands; 5 Centre for Population Screening, RIVM Dutch National Institute for Public Health and Environment, 3720 BA Bilthoven, The Netherlands

**Keywords:** neonatal screening, questionnaire, attitudes, pregnancy, guidance, maternity, information, education, professionals, midwives

## Abstract

Newborn bloodspot screening (NBS) aims to detect treatable disorders in newborns to offer early interventions. According to the official Dutch national NBS guidance, parents in the Netherlands should be informed about NBS during pregnancy by maternity care providers (MCPs), providing two leaflets and oral information. This study investigated what, how, and when information about NBS is given during pregnancy according to Dutch MCPs. An online questionnaire was completed by 279 MCPs; 237 (84.9%) provided information to parents themselves, although 4.6% of them only did so postnatally, and 240 (86.0%) considered this the task of the MCP. Among the 237 MCPs, information was provided by personal conversation (59.9%) and by giving at least one leaflet (83.1%), while 25.7% only gave leaflets. Being a first pregnancy (45.1%) and parents’ literacy (38.8%) influenced how MCPs provided information. Information was mostly provided at 34–37 weeks gestation (68.8%). Conversations mostly included giving information on when NBS will be performed (97.2%), the purpose of NBS (93.7%), how the test will be performed (92.3%), and participation being voluntary (80.3%). The results suggest that while most Dutch MCPs consider it their task to provide NBS information, its timing, method, and completeness do not always follow the established guidelines.

## 1. Introduction

Newborn bloodspot screening (NBS) aims to detect treatable conditions early in order to quickly initiate appropriate interventions to prevent or minimize the impact on the child’s health and development. Information provision to parents about NBS is recognized as an integral part of the NBS process [1]. Where consent is required, parents decide on behalf of their newborn. Providing sufficient information to parents about NBS, including the purpose and consequences of screening, is considered important to enable them to make an informed decision about participation [2]. Moreover, understanding NBS allows them to practically prepare for the process so they know what to expect and are prepared for possible outcomes. Literature review shows that many parents are not fully informed about NBS, including the conditions screened, the implications of positive results, and the possible scientific use of dried blood spots [3]. Lack of information may increase anxiety and stress associated with positive NBS results [4]. Parents’ understanding is also becoming increasingly important given the expansion of screening programs to include more conditions, as a lack of understanding may contribute to reduced acceptance of NBS and increased parental anxiety [5,6].

Information provision on NBS varies per country, including the level of detail of the information as well as how it is provided and when [2]. Parents are generally informed of the heel prick test at the time of blood specimen collection. Offering information during pregnancy can, however, provide timely and more layered information to parents. Moreover, parents themselves mention that they prefer to have information during pregnancy instead of only after birth [5,7,8,9]. A focus group study in the United States (US) that included parents showed that participants believed that parents should receive the information preferably in the third trimester of pregnancy [7]. In addition, the American College of Obstetricians and Gynecologists recommends that obstetric care providers should make resources about NBS available to patients during pregnancy [10].

Little is known about the perspectives of maternity care providers (MCPs) towards information provision during pregnancy. A United Kingdom survey study showed that midwives preferred to provide the information late in pregnancy to improve parents’ ability to make a decision about NBS [11]. A US survey study in the early 2000s showed that there is a gap between what MCPs say they do and what they are supposed to do with regard to informing parents about NBS [12].

In the Netherlands, NBS is voluntary, though few parents decline. In 2022, the uptake was 98.9%, with about 167,000 children screened for 26 conditions [13]. According to the official Dutch NBS quality guidance, parents should be informed of the screening process at different moments, including during the first and third trimesters of pregnancy and after birth [14]. This study investigated what, how, and when information about NBS is actually given during pregnancy according to Dutch MCPs.

## 2. Materials and Methods

### 2.1. Design and Ethics Statement

An online cross-sectional survey was used among practicing MCPs in the Netherlands. Participants ticked a box before starting the questionnaire confirming that they gave their informed consent to participate in the study. Answers were collected anonymously. The study protocol (no. 2022.0107) was reviewed by the Medical Ethical Committee of Amsterdam University Medical Centers. The committee concluded that the act of medical research involving human subjects (WMO) did not apply and therefore exempted the protocol from needing further approval.

### 2.2. Study Population

Several strategies were used to identify and approach practicing MCPs. Firstly, e-mail addresses of relevant maternity care organizations were collected using the website of the national Perinatal Care College (CPZ) (www.kennisnetgeboortezorg.nl, accessed on 15 February 2022), contact forms on the websites of midwifery consortia, and the researchers’ networks. Secondly, a link with information about the study and the questionnaire was shared using social media (LinkedIn, Instagram, and Facebook). Finally, seven regional consortia, the network of the Royal Dutch Organization for Midwives (KNOV), and 61 midwifery–obstetric partnership networks (VSV) were sent an e-mail requesting them to share the information about the study and the questionnaire link with practicing MCPs. Data collection was conducted between March and May 2022. Participants were included if they were currently working in the Netherlands as MCPs, including midwives and obstetricians.

### 2.3. Setting: NBS in the Netherlands

In the Netherlands, the Dutch National Institute for Public Health and the Environment Centre for Population Screening (RIVM-CvB) directs, manages, and coordinates NBS. A quality guidance resource describing the screening standards is provided to all professionals involved in the execution of the screening, including MCPs. This resource includes guidance for MCPs for providing standardized information on NBS to future parents [14].

The RIVM-CvB guidance resource also states at what gestational age information should be delivered by the MCP and what information should be included. During the first trimester of pregnancy (first consultation), the MCP, usually a community midwife, should hand out the leaflet “Pregnant!” which discusses more general aspects of pregnancy and includes NBS. This involves one brief paragraph on the general aspects of NBS (timing and purpose) with a QR code to a short online video that illustrates the procedure. During the third trimester, the MCP should inform the parents on NBS orally during a consultation as part of the informed consent procedure, after which the second leaflet, “Heel prick and hearing test in newborns”, is handed out. During the consultation, the MCP can use a checklist that requires information to be given about the purpose of NBS, general information (procedure, possible outcomes, and privacy issues), consent for scientific use of data, organization and process, general information about the conditions, and the possibility of being reported on carrier status for sickle cell disease [14]. MCPs can direct parents to a website (www.pns.nl/hielprik, accessed on 1 March 2022), with information available in ten languages.

Information is also provided right before the screening is conducted (the heel prick should be carried out between 72 and 168 h after birth), either at home by a nurse, midwife, or a youth healthcare worker or in a hospital by a hospital healthcare worker.

### 2.4. Questionnaire

The questionnaire used in the study was developed based on the RIVM-CvB guideline for NBS screening. Readability and accuracy of the questionnaire were evaluated by two RIVM employees (R.V.P. and E.E.). Microsoft Forms was used to design the questionnaire. A translated version of the questionnaire can be found in the Supplementary Materials. Measures included respondents characteristics to assess current work status as an MCP, sex, age category, years of working experience as an MCP, work setting, practice characteristics, province of employment, and country of education.

The questionnaire included questions on how and when information is provided and on the content of information provision.

*How and when information is provided*. Respondents were asked who informs the parents about NBS in their practice or hospital and whether (oral or written) information on NBS was provided. An open question was added to elaborate on the answers given. Respondents were also asked what factors or characteristics of the pregnant parents influenced the way of providing information (none, literacy, migrant background, education level, socioeconomic status, and parity). One question assessed how information about NBS was provided. A sum score of the different methods (e-mail, leaflets, website, and consultation) was calculated to help determine how many of these methods were used by the respondents in their information provision. Respondents were asked when (weeks of gestation) the information is provided during pregnancy or if information is being provided only postnatally. Respondents were finally asked whether they thought that it was the task of the MCP to provide information about NBS and were asked to explain their answer.

*Content of information provision (what).* One question assessed information provision about NBS to parents in personal conversation. In addition, respondents were asked whether they ever get questions from pregnant parents about NBS and were asked to elaborate if they chose Yes.

### 2.5. Statistical Analyses

Answers to open questions were coded using content analysis by J.E.K. and discussed with L.H. until agreement was reached. Descriptive statistics were used to describe the characteristics. Chi-square analysis was used to compare subgroups based on participant work setting for the question, “Do you think it is the task of the MCP to inform women about NBS?”. A *p* value < 0.05 was considered to indicate statistical significance. All quantitative analyses were conducted using SPSS 28.0 (IBM Statistics for Windows, IBM, Armonk, NY, USA).

## 3. Results

### 3.1. Respondents’ Characteristics

The online survey was completed by 285 respondents. In the Netherlands, there are about 3000 primary care midwives. The actual response rate could not be calculated due to the survey distribution methods. A total of 6 respondents were excluded from analyses because they did not meet the inclusion criteria, leaving 279 for analyses. Respondents’ characteristics are summarized in Table 1. Most of the MCPs (61.3%) had more than 10 years of work experience in their field, were community midwives (85.7%), and were working in the western part of the Netherlands (50.5%). The sample was mainly educated in the Netherlands (85.6%).

### 3.2. How and When Information Is Provided

Table 2 shows that 86.0% of MCPs believed it was the task of the MCP to provide information about NBS, while 14% (n = 39) did not think it was their task. Community midwives were more likely to report that it was their task compared to respondents working in other settings (87.9% versus 75%, *p* = 0.03). Most MCPs who did not believe it was their task explained in the open text field that they were not paid for it and believed it was the responsibility of the organization or person taking the sample. In total, 237/279 MCPs (84.9%) reported they provide information to pregnant women themselves during regular consultation, 11.5% reported that someone else in or outside of the practice informs pregnant women, and 3.6% did not know who provides the information.

The majority of the 237 MCPs who provide information (212, 89.5%) reported they always provide oral and/or written information, whereas 25/237 (10.5%) of these MCPs reported that they do not always do this. Analysis of open answers revealed that this mostly depends on the MCP’s available time and parity. Parity was also the most commonly mentioned characteristic of women that influenced how information is given by MCPs (45.1%), with less information provided in the case of multiparity. Other factors frequently mentioned were women’s literacy (38.8%) and having a migration background (31.2%). In total, 93 MCPs (39.2%) reported informing every woman in the same way (Table 2).

Leaflets were the most common way of information provision on NBS during pregnancy; 75.9% gave the leaflet “Heel prick and hearing test in newborns”. In total, 179/237 MCPs (83.1%) gave at least one leaflet, and 46.4% gave both leaflets. Of the 237 MCPs, 25.7% (n = 61) only gave leaflets (one or both leaflets). Information was provided through personal conversation by 142 of 237 (59.9%) MCPs, with the majority of these MCPs (127/142, 89.4%) using the leaflet “Heel prick and hearing test in newborns” during the conversation. A minority referred to the website (19.8%). A total of 93/237 (39.2%) used one way to inform women (e-mail, at least one leaflet, website, or personal conversation), 39.7% used two different ways, 16.5% used three ways, and 3% used four ways.

The most frequently reported period to provide information was at 34–37 weeks gestation (163/237, 68.8%), with 26/237 (11%) giving information before 27 weeks and only 7.2% giving information in the first trimester. Notably, 11/237 (4.6%) reported only providing information during the postnatal period.

### 3.3. Content of Information Provision (What)

Table 3 shows that, of the 142 MCPs that provide information during personal conversation, the information most commonly addressed included when the screening is performed (97.2%), the purpose of the screening (93.7%), how the test is performed (92.3%), and the screening being voluntary (80.3%). Few mentioned the overall accuracy of the screening (7.7%) or addressed every condition being tested for (2.8%).

Of the 279 MCPs, 104 (37.4%) reported that they sometimes receive questions about NBS from pregnant women. Frequently mentioned examples of questions that these MCPs receive included practical questions (e.g., who will perform the heel prick, when is the test performed, etc.), what kind of conditions are included in NBS, and whether the test is mandatory.

## 4. Discussion

This survey study among maternity care practitioners in the Netherlands shows that most respondents provide information on NBS to women during pregnancy and also consider this a task of the MCP. Information was mostly given orally and offered at 34–37 weeks gestation, while about one in four MCPs gave only leaflets. Personal conversations usually included giving information on when and how the screening will be performed, the purpose of screening, and participation being voluntary.

A minority of maternity care providers, and especially those who were not community midwives, did not think it was the MCP’s task to provide information to parents, although this is defined in the Dutch national guidelines [14]. An earlier survey among US providers showed that midwives were more likely to perceive professional responsibility to inform parents compared to obstetricians [15]. Moreover, it showed that professionals who perceive a responsibility to inform parents were more likely to do so [15]. Lack of confidence, knowledge, educational materials or training, and workload can be seen as barriers to MCPs giving adequate explanations to parents about NBS [12,15,16].

Sixty percent of our respondents who provided information did so in personal conversation, whereas the guidance requires that all parents be informed in this way. A United Kingdom (UK) internet panel study showed that (future) parents preferred to discuss the information in a face-to-face meeting and receive information before the child is born [8]. The results of that study also suggested that increasing the number of conditions in NBS, as is currently the case in most countries, would lead to more parents being averse to receiving the information (only) at the time of testing [8].

Our results show that MCPs often adjust the information provision to parental characteristics, such as parity and, to a lesser extent, to parents’ literacy or migration background. Our study does not reveal exactly how this information is adapted by MCPs to parents’ backgrounds or what is considered helpful in this regard. In the US, it has been shown that parents in medically underserved areas less often recall being informed before birth compared to parents living outside of these areas, suggesting more distinct methods are needed to reach these parents [17]. A randomized controlled trial using an educational intervention, including an NBS movie and brochure, in the third trimester of pregnancy, positively influenced women’s attitudes towards NBS [18]. Online multimedia tools for parents, including video, might thus be helpful to improve educational efforts, especially for parents in medically underserved areas [17].

According to the Dutch RIVM-CvB guideline, information should be given at two instances in pregnancy: by handing out a leaflet (“Pregnant!”) in the first trimester as a first introduction to the topic and in the third trimester by an oral consultation accompanied by a more comprehensive leaflet. In our study, most MCPs provided information in the third trimester, which is generally considered the most optimal time according to parents and professionals [7,11,19], while few reported giving information before 27 weeks. A UK survey study of professionals’ preferences showed that midwives did not favor providing information before 20 weeks gestation when asked about their preferred timing because it was seen as limiting parents’ ability to make a decision [11]. Offering information at different times can give parents more time to process a greater amount of information, especially if this is new information.

Globally, parents are informed differently about NBS. A 2018 study comparing written parental information products from 26 European countries found that all products included information on the purpose of screening, but only eight included information on the possibility of false-positive and false-negative findings [2]. In our study, very few MCPs discussed the overall accuracy of the screening. In line with this, a UK National Health Services survey found that midwives believed that all types of information would improve parents’ decision-making for NBS, except for the possibility of receiving false-positive results [11]. A parallel survey among parents, however, suggested that as the number of conditions increases, the possibility of getting false-positive results becomes a more important piece of information for parents [8]. In general, it is important for parents to understand the uncertainties of a screening test and that diagnostic testing is necessary to confirm positive results [2].

The findings may be useful in developing additional studies to better understand current information provision, including parents’ views on the provision of information about NBS during pregnancy. For example, a recent survey found that only 47% of Dutch parents reported being well informed about NBS during pregnancy [5]. MCPs can be seen as the professionals who direct parents to the relevant sources of information and are first informants about NBS, providing accurate and complete information. MCPs should therefore check parents’ understanding of NBS and tailor information to parents’ needs and literacy levels, which, according to the findings, currently seems to be largely lacking. In addition to the initial training of MCPs, continued attention is needed to educate MCPs on the topic and address their roles and responsibilities, such as through ongoing professional development via e-learning.

### Limitations

This study had some limitations. Due to the way of recruitment, the response rate could not be calculated. Most respondents were midwives working in the western part of the country. Less than 10% of all primary care midwives participated in the study. Subgroup analyses between participants from different backgrounds were limited due to low numbers. Selection bias of those most interested in the topic and most likely to provide educational materials to parents is possible. The results also reflect respondents’ descriptions of their practice, but we do not know how this compares to actual practice.

## 5. Conclusions

The findings show that although most MCPs who participated in the study inform parents about NBS during pregnancy and consider this the task of the MCP, this is not always provided according to the Dutch national guidance. The timing, method, and completeness of information about NBS provided by MCPs need improvement to more effectively engage them in adequately informing and preparing parents for newborn screening. This is crucial not only for obtaining informed consent where required but also for sustaining parents’ acceptance of NBS, particularly as programs continue to expand to monitor more conditions.

## Figures and Tables

**Table 1:** Respondents’ characteristics.

	Maternity Care Providers n = 279 n (%)
Sex	
Female	275 (98.6)
Male	4 (1.4)
Age group	
20–30 years	70 (25.1)
31–40 years	97 (34.8)
41–50 years	67 (24.0)
>50 years	45 (16.1)
Work experience	
<2 years	15 (5.4)
2–5 years	52 (18.6)
6–10 years	41 (14.7)
>10 years	171 (61.3)
Work setting	
Community midwife	239 (85.7)
Clinical midwife	25 (9.0)
Obstetrician (or resident)	13 (4.7)
Nurse	2 (0.7)
Characteristic of practice (37 missing)	
Group with one team	166 (68.6)
Group with multiple teams	40 (16.5)
Duo	24 (9.9)
Caseload	12 (5.0)
Working region of the Netherlands	
Northern	30 (10.8)
Eastern	41 (14.7)
Southern	67 (24.0)
Western	141 (50.5)
Country of education (1 missing)	
The Netherlands	238 (85.6)
Belgium	36 (12.9)
Other	4 (1.5)

**Table 2:** How and when information about NBS is provided during pregnancy.

	Maternity Care Providers n (%)
**Do you think it is the task of the MCP to inform women about NBS?**	n = 279
Yes	240 (86.0)
No	39 (14.0)
**Who informs pregnant women about NBS in your practice or hospital?**	n = 279
I give information about the screening during a regular consultation	237 (84.9)
Someone else in (or outside of) the practice informs the pregnant woman	32 (11.5)
I do not know	10 (3.6)
**What factors influence how information is given (multiple responses)**	n = 237
Parity	107 (45.1)
There are no factors influencing the way information is given; I inform everyone in the same way	93 (39.2)
Literacy of the pregnant woman	92 (38.8)
Migration background	74 (31.2)
Level of education	56 (23.6)
Socioeconomic status	26 (11.0)
**How is information given (multiple responses)?**	n = 237
By giving the leaflet “Heel prick and hearing test in newborns”	180 (75.9)
By giving the leaflet “Pregnant!”	127 (53.6)
By personal conversation about the screening, where I DO use the leaflet “Heel prick and hearing test in newborns”	114 (48.1)
By referring to the website www.pns.nl, accessed on 1 March 2022 (orally or via e-mail)	47 (19.8)
By standardized e-mail	42 (17.7)
By personal conversation about the screening, where I DO NOT use the leaflet “Heel prick and hearing test in newborns”	28 (11.8)
By personal e-mail	7 (3.0)
**When is information given (multiple responses)?**	n = 237
<18 weeks gestation	17 (7.2)
18–27 weeks gestation	9 (3.8)
28–33 weeks gestation	74 (31.3)
34–37 weeks gestation	163 (68.8)
38–42 weeks gestation	13 (5.5)
The information is not given during pregnancy, only after birth	11 (4.6)

NBS, newborn bloodspot screening; MCP, maternity care provider.

**Table 3:** Content of information provided in personal conversation.

	Maternity Care Providers n (%)
What are pregnant women or couples told about NBS? (multiple responses)	n = 142
When the screening is performed	138 (97.2)
Purpose of the screening	133 (93.7)
How the test is performed	131 (92.3)
That the screening is voluntary	114 (80.3)
When the results can be expected	107 (75.4)
That the screening can find carriers (of sickle cell)	96 (67.6)
How many conditions are tested	91 (64.1)
Some of the conditions being screened for, namely the most common conditions	78 (54.9)
That NBS is part of population screening	66 (46.5)
The option to save blood spots for scientific research	56 (39.4)
Overall accuracy of the screening	11 (7.7)
Every condition being tested for	4 (2.8)
Practically nothing	0 (0)

## Data Availability

The data presented in this study are available on reasonable request from the corresponding author. The data are not publicly available due to privacy restrictions.

## Supplementary Materials

The following supporting information can be downloaded at: https://www.mdpi.com/article/10.3390/ijns11010005/s1, Questionnaire S1.
