# Supplementary material for: Maternity Care Providers’ Experiences with Providing Information on Newborn Bloodspot Screening During Pregnancy: A Dutch Survey Study
Source: Int J Neonatal Screen. 2025 Jan 8;11(1):5. doi: 10.3390/ijns11010005 (PMC11755565; doi:10.3390/ijns11010005)
Supplement: Supplementary file 1 [file IJNS-11-00005-s001.zip › IJNS-3379548-supplementary.pdf]

Supplementary Material

Translated questionnaire "Maternity care providers' experiences with providing information on newborn bloodspot screening during pregnancy"

1. Do you want to participate in this study?

☐ Yes
☐ No (*exclude*)

2. Are you currently working as a maternity care provider?

☐ Yes
☐ No
☐ Other namely:

3. What is your sex?

☐ Male
☐ Female
☐ Other
☐ I do not want to answer

4. Which age group do you belong to?

☐ 20 - 30 years
☐ 31 - 40 years
☐ 41 - 50 years
☐ 51 - 60 years
☐ > 60 years

5. How many years of work experience do you have in obstetrics/maternity care?

☐ < 2 years
☐ 2 - 5 years
☐ 6 - 10 years
☐ 11 - 15 years
☐ > 15 years

6. In which setting are you working?

☐ Midwife in training
☐ Primary care midwife
☐ Clinical midwife in the hospital
☐ Gynecologist in the hospital
☐ Medical resident gynecology in the hospital
☐ Other namely:

7. What is the characteristic of your practice?

☐ Caseload practice
☐ Duo practice
☐ Group practice with one team
☐ Group practice with multiple teams

8. In which province are you working (mainly)?

☐ Groningen
☐ Friesland

☐ Drenthe
☐ Flevoland
☐ Overijssel
☐ Gelderland
☐ Utrecht
☐ Noord-Holland
☐ Zuid-Holland
☐ Zeeland
☐ Noord-Brabant
☐ Limburg

9. What is your country of education?
☐ Netherlands
☐ Belgium
☐ Other namely:

Information provision about Newborn Screening (NBS)
10. Is (oral or written) information about NBS provided in your practice or hospital?
☐ Yes, always
☐ Yes, but not always
☐ No
☐ I do not know
☐ It depends
☐ Other namely:
Can you elaborate on your answer?

11. How is information about this screening generally provided in your work setting? Multiple answers are possible.
☐ By personal e-mail
☐ By standardized e-mail
☐ By giving the leaflet “Pregnant!” by the RIVM
☐ By giving the leaflet “Heel prick and hearing test in newborns” by the RIVM
☐ By referring to the RIVM website (www.pns.nl; also applicable when referred to the website via e-mail)
☐ By personal consultation about the screening, where I DO NOT use the leaflet “Heel prick and hearing test in newborns”
☐ By personal consultation about the screening, where I DO use the leaflet “Heel prick and hearing test in newborns”
☐ Other, namely:

12. Who informs the pregnant woman about NBS in your practice or hospital?
☐ I give information about the screening during a regular consultation
☐ Someone else in the practice informs the pregnant woman
☐ I do not know
☐ Other namely:

13. What are pregnant women or couples told about NBS in your setting? Multiple answers are possible.
☐ Practically nothing
☐ How many conditions are tested for
☐ Every condition being tested for

☐ Some of the conditions being tested for, namely the most common conditions
☐ Purpose of the screening
☐ That the screening is voluntary
☐ That NBS is part of population screening
☐ How the test is performed
☐ When the screening is performed
☐ When the results can be expected
☐ Overall accuracy of the screening
☐ That the screening can also find carriers of sickle cell disease
☐ The option to save blood for scientific research
☐ Other, namely:

14. When during the pregnancy is information about NBS provided? Multiple answers are possible.
☐ < 18 weeks
☐ 18 – 27 weeks
☐ 27 - 30 weeks
☐ 31 – 33 weeks
☐ 34 – 37 weeks
☐ 38 – 42 weeks
☐ The information is not given during pregnancy, only after birth
☐ Other, namely:

15. Which factors in your work setting or characteristics of pregnant women influence how you provide information about NBS? Multiple answers are possible.
☐ There are no factors that influence the way information is provided, I inform everyone in the same way
☐ Literacy of the pregnant woman
☐ Having a migration background or not
☐ Level of education
☐ Social economic status
☐ Parity
☐ Other, namely:
Can you elaborate on your answer?

16. RIVM supplies the leaflet "Heel prick and hearing test in newborns" and the website www.pns.nl/hielprik. How can the RIVM improve these communication tools to better support maternity care providers in providing information about NBS?

17. Do you think it is the task of the maternity care providers to provide information about NBS?
☐ Yes
☐ No
Can you elaborate on your answer?

19. Do pregnant women ask you questions about NBS?
☐ No
☐ Yes, namely:

Anything else I would like to say about NBS information provision?
Thank you for your participation!
